# Evaluating the psychometric properties of the Chinese Depression Anxiety Stress Scale for Youth (DASS-Y) and DASS-21

**DOI:** 10.1186/s13034-023-00655-2

**Published:** 2023-09-07

**Authors:** Cui-hong Cao, Xiao-ling Liao, Jeffrey H. Gamble, Ling-ling Li, Xing-Yong Jiang, Xu-Dong Li, Mark D. Griffiths, I-Hua Chen, Chung-Ying Lin

**Affiliations:** 1 School of Foreign Languages, Shandong Women’s University, Jinan, 250300 China; 2 Faculty of Education, Qufu Normal University, Qufu, 273165 China; 3 Faculty of Education, Jiangxi Science and Technology Normal University, Nanchang, 330031 China; 4 Department of English, National Changhua University, Changhua, 50007 Taiwan; 5 Xinjian District of Nanchang City, No.1 Senior High School, Nanchang, 330100 China; 6 Yangan Primary School of Qionglai City, Qionglai, 611535 China; 7 Gaogeng Nine-year School, Qionglai, 611533 China; 8 International Gaming Research Unit, Psychology Department, Nottingham Trent University, Nottingham, UK; 9 Chinese Academy of Education Big Data, Qufu Normal University, Qufu, 273165 China; 10 Institute of Allied Health Sciences, College of Medicine, National Cheng Kung University, Tainan, 701401 Taiwan; 11 Department of Occupational Therapy, College of Medicine, National Cheng Kung University, Tainan, 701401 Taiwan; 12 Biostatistics Consulting Center, National Cheng Kung University Hospital, College of Medicine, National Cheng Kung University, Tainan, 701401 Taiwan; 13 Department of Public Health, College of Medicine, National Cheng Kung University, Tainan, 701401 Taiwan

**Keywords:** Psychometric properties, Children, Adolescents, DASS-Y, DASS-21

## Abstract

**Background:**

In recognizing the need for a reliable and valid instrument to assess psychological distress among children and adolescents, the present study translated the newly developed Depression Anxiety Stress Scale for Youth (DASS-Y) into Chinese, while also comparing its psychometric properties with those of the well-established DASS-21 within a primary and middle school demographic.

**Methods:**

Utilizing a combination of convenience sampling and purposive sampling, a cohort comprising 1,507 primary and 1,131 middle school students was recruited. Rasch analysis, confirmatory factor analysis (CFA), and structural equation modeling (SEM) were used in the data analysis.

**Results:**

Significant differences were observed between the DASS-Y and the DASS-21, notably within the anxiety subscale. The proportions of individuals with clinical mood disorders identified by the two scales demonstrated a significant disparity. Both scales, following an adjustment of responses, exhibited satisfactory internal consistency and convergent validity, with the acceptance of a three-factor structure. Furthermore, the DASS-Y showed superior discriminant validity relative to the DASS-21, providing more compelling evidence regarding concurrent validity.

**Conclusion:**

Overall, the Chinese version of the DASS-Y demonstrated superior robustness to the DASS-21 in terms of psychometric properties. The findings provide initial evidence for the psychometric properties of the DASS-Y from another culture.

**Supplementary Information:**

The online version contains supplementary material available at 10.1186/s13034-023-00655-2.

## Introduction

Mental health disorders affect approximately 14% of 10- to 19-year-olds worldwide [1]. Most of these go unrecognized (and therefore untreated) representing 13% of the global burden of disease according to the World Health Organization [1]. Mental health problems among children and adolescents can negatively impact their physical health [2], academic performance [3] and social lives [4]. They can also lead to substance abuse [5], self-harm, and suicidal behavior [5, 6]. Mental health disorders not only occur at younger developmental stages but also continue to exist and influence their later life [4, 7–9]. Of more concern is that mental health problems appear to be on the rise [10]. With this in mind, assessing the mental health states of children and adolescents is a prerequisite for developing and implementing prevention and intervention. Therefore, a reliable and valid instrument specifically used for assessing the mental health states of this population is crucial and urgently needed.

The Depression Anxiety Stress Scale for Youth (DASS-Y) is a newly developed instrument for comprehensively assessing the negative affect of depression, anxiety and stress among both children and adolescents [11]. The DASS-Y is the youth version of the Depression Anxiety Stress Scale (DASS) which was published in April 2022 by Szabó and Lovibond whose team have engaged in a series of studies in this area since 2006 [11–14]. The full DASS consists of 42 items which are divided into three self-report subscales that assess depression, anxiety, and stress symptoms [15]. In addition to the full 42-item version, there are also 21-item (DASS-21) [16], 12-item [17], 9-item [18], and 8-item [19] shortened versions. For each subscale, the higher the total score, the more severe the symptom is reported to be. Psychometric properties of all the versions have been supported by a significant amount of published empirical evidence [20–25]. The DASS has been validated in clinical [20, 21] and nonclinical samples [22, 23], and in more than 50 different language versions [26], including Nepalese [24, 25], Arabic [27], Persian [28], Maltese [29], Turkish [30], Chinese [31, 32], Brazilian Portuguese [33], and among different age groups (e.g., 60 years and older [24], 40 years and older [34], from 14 to 55 years [35], and emerging adults [36]).

With its ease of completion and scoring, the DASS-21 has been extensively studied and supported for its satisfactory discriminant validity in assessing adults’ depression, anxiety, and stress [37–39]. However, there are doubts about whether this measure originally developed for adults can be applied to children and adolescents [16, 40, 41], as well as controversies concerning its factor structure [42, 43]. Szabó [13] explored the factor structure of the DASS-21 in a young adolescent sample and found that the constructs of depression and anxiety in adults are similar to those in adolescents at the age of 11 to 15 years whereas the construct of stress was doubtful in the younger age group. Hashim et al. [44] examined the factorial validity of the DASS-21 among Malaysian adolescents and found that this scale was best used as a one-factor model to assess general negative affect among adolescents. Shaw et al. [16] supported the bifactor structure (i.e., a general factor and three specific subscales) and concluded that the DASS-21 could be applied to assessing general distress among adolescents. However, it was unable to distinguish the separate states of depression, anxiety and stress in this age group. Mellor et al. [45] supported the three-factor structure of the DASS-21 when it was applied to adolescents from high schools in four different cultural contexts (i.e., Australia, Chile, China and Malaysia).

With regard to previous studies, several assert that the existing versions of DASS targeted at the adult population may not be applicable to assessing psychological distress among children and adolescents [16, 41]. The reasons can be summarized as follows: the poor readability of the items of the self-report measures, youth’s lower interpretation ability and not being able to distinguish depression, anxiety, and stress [16], and their comprehension and interpretation problems of the terms used in the items [14]. Considering the current inapplicability of the adult DASS to children and adolescents, it highlights the need for a self-report scale like DASS for youth.

To address this issue, the DASS-Y was very recently developed and validated among children and adolescents aged 8–17 years [11]. The development of the youth version of the DASS underwent psychometric evaluation. In the beginning, with the purpose of verifying whether depression, anxiety, and stress identified by the DASS among adults were also present in children, Szabó and Lovibond [12] created 76 items based on the State-Trait Anxiety Inventory for Children–Trait Version, the Children’s Depression Inventory, and broad item content of the core symptoms defined by the DASS. They found that anxiety and stress could not be distinguished. Szabó and Lovibond [12] speculated this was largely limited by expression of some item content and recommended that instead of being accepted directly from adult scales, every item should be evaluated to make it completely understandable by youth.

Following this, Fowler and Szabó [14] utilized 36 modified DASS items through retaining, adapting, and deleting items in order to make them more easily understood. Their aim was to investigate whether stress identified among adults could similarly be evaluated among youth. The study identified three separate factors analogous to depression, anxiety, and stress in the adult DASS. They pointed out that failure to recognize stress as an independent emotional symptom resulted from problems of understanding, and suggested further refinement of expression in some items as needed. In 2022, and guided by previous research, Szabó and Lovibond [11] developed the 21-item DASS-Y based on 40 draft items. They showed it was psychometrically sound when assessing depression, anxiety and stress among youth aged 8–17 years using confirmatory factor analysis (CFA) and multiple regression analysis. They concluded that the final 21-item three-factor scale was well suited for both children and adolescents.

Given that the DASS-Y has recently been developed, only Szabó and Lovibond’s original study [11] has tested the psychometric properties of its English version (among Australian children and adolescents). Therefore, there is scant evidence regarding its psychometric properties in other cultures and languages. Consequently, the present study translated the DASS-Y into Chinese, evaluated it, and compared its (and the DASS-21’s) psychometric properties among Chinese children and adolescents using both classical test theory as well as Rasch analysis, including item diagnosis, factorial, convergent, and discriminant validity. Classical test theory, which is commonly adopted to examine psychometric properties of scales, can assist in validating the theoretical structure, and resolve the factorial validity controversy of the scale [46, 47]. However, it does not provide a precise description of the scale in terms of item-level properties [48]. Therefore, in order to be confident that an instrument is psychometrically robust, it is better to be tested using a variety of statistical methods [49]. Rasch analysis, which evaluates how well each item and its category fits within a mathematical model [48], can contribute additional insight to the psychometric properties of the DASS-Y and DASS-21, and complement the results of the classical test theory. Additionally, combining the two psychometric testing methods together has previously been applied to evaluating the psychometric properties of the DASS-21 [50, 51]. In this regard, the combination of these two analytical approaches to evaluate the newly developed DASS-Y provides more comprehensive psychometric test evidence.

In addition, the concurrent validity of the two scales was assessed through their correlation with emotional exhaustion, a primary element of academic burnout [52]. This approach is guided by two key rationales. Firstly, it adheres to the widely accepted Stressor–Strain–Outcome (SSO) model. In this model, emotional exhaustion frequently symbolizes the strain that leads directly to psychological distress as an outcome [52]. Secondly, this relationship is backed by empirical research showing a close association between emotional exhaustion and psychological distress in primary and middle school students [53, 54]. Notably, Salmela-Aro et al. [53] carried out two longitudinal studies which demonstrated that school burnout robustly predicted symptoms of depression, emphasizing the influence of burnout on student depression. Similarly, Gungor [54] reported that school-induced emotional exhaustion had negative impacts on the mental health of Turkish middle school students. Furthermore, Shin et al. [55] investigated the relationship between mental health problems and academic burnout among Korean middle school students, and found that depression and anxiety were particularly associated with the exhaustion aspect of academic burnout. In light of both the theoretical framework of the SSO model and the aforementioned empirical evidence, emotional exhaustion was chosen as the criterion for assessing the concurrent validity of the two scales under examination.

## Materials and methods

### Participants and procedures

The present study used a combination of convenience sampling and purposive sampling to recruit primary and middle school students. Data collection lasted for two months from November, 2022 to December, 2022. Ethical approval was obtained in advance from the Institutional Review Board (IRB) of Jiangxi Psychological Consultant Association (IRB ref: JXSXL-2022-CL15). To collect sufficient data, the research team initially liaised with principals from primary schools in two southwestern Chinese cities and middle schools in central provincial capitals, seeking their assistance in administering an online survey to their students. Finally, five primary and two middle schools agreed to distribute the survey. Convenience sampling allowed for easier recruitment of participants in the target group in a less time-consuming and more cost-effective manner [56, 57]. However, the study also adopted purposive sampling to ensure the participants met the specific criteria for inclusion. The inclusion criteria were: (i) being a student from fourth grade or higher in a primary school or in a middle school; (ii) being able to understand written Chinese; and (iii) having parents who agreed to their child’s participation. The implementation process commenced with class tutors clearly explaining the survey’s purpose to the students. They also informed students of their rights, including voluntary participation and data privacy. Next, teachers sent the survey link to the students’ parents via mobile devices. Parents who agreed to participate in the present study, then voluntarily supervised their children’s completion of the survey at home. The website’s opening page contained a consent form, which required agreement before officially beginning the survey. The total number of valid responses received was 1,507 from primary school students and 1,131 from middle school students. For the online survey to be submitted, all items needed to be answered. Therefore, there were no missing data.

### Measures

The present study used the DASS-21, DASS-Y, and the Maslach Burnout Inventory–Student Survey. The following sections delineate how these tools are scored and elucidate the nature of their internal consistencies. Given that both the DASS-21 and DASS-Y inherently contain three subscales, they are multidimensional. To address this, both McDonald’s Omega total (*ω*_t_) and McDonald’s Omega Hierarchical (*ω*_*h*_) in these two scales are reported [58].

#### The Depression Anxiety Stress Scale–21-item version (DASS-21)

The DASS-21 is a shortened version of the full 42-item DASS developed by Lovibond and Lovibond [15]. The self-report instrument comprises three subscales (i.e., depression, anxiety, and stress), with seven items per subscale. Each item is rated on a four-point scale to rate the extent to which participants have undergone the emotional state over the past week from 0 (*not true*) to 3 (*very true*). Example items from the three subscales are: “*I couldn’t seem to experience any positive feeling at all*” (depression), “*I was aware of dryness of my mouth*” (anxiety), and “*I tended to over-react to situations*” (stress). The internal consistency of the DASS-21 subscales, as evaluated in the present study, exhibited acceptable levels. Among the primary school participants, the depression subscale had a *ω*_*t*_ of 0.91 and a *ω*_*h*_ of 0.84. The anxiety and stress subscales had a *ω*_*t*_ of 0.89 and *ω*_*h*_ of 0.81, respectively. Similarly, the middle school participants had comparable patterns. The depression subscale had a *ω*_*t*_ and a *ω*_*h*_ of 0.89 and 0.84, respectively. The anxiety subscale had a *ω*_*t*_ and a *ω*_*h*_ of 0.85 and 0.81, while the stress subscale had a *ω*_*t*_ and *ω*_*h*_ of 0.84 and 0.76.

#### The Depression Anxiety Stress Scale for Youth (DASS-Y) and translation procedure

Developed and validated by Szabó and Lovibond in 2022 [11], the DASS-Y is the youth version of the DASS, comprising simplified wording. Similar to the original DASS-21, it also comprises 21 items equally divided into three subscales (i.e., depression, anxiety and stress). The DASS-Y adopts the same scoring approach as the original DASS with all the items scored on a four-point Likert scale. Example items in the three subscales are: “*I felt that life was terrible*” (depression), “*I felt scared for no good reason*” (anxiety), and “*I was easily annoyed*” (stress). In the present study, the internal consistencies of the DASS-Y subscales demonstrated satisfactory levels. Among the primary school participants, the depression subscale had a *ω*_*t*_ and *ω*_*h*_ of 0.89 and 0.85, respectively. The anxiety subscale had a *ω*_*t*_ and *ω*_*h*_ of 0.89 and 0.75, while the stress subscale had a *ω*_*t*_ and *ω*_*h*_ of 0.89 and 0.79. Similarly, among middle school participants, the depression subscale had a *ω*_*t*_ and *ω*_*h*_ of 0.84 and 0.85. The anxiety subscale had a *ω*_*t*_ and *ω*_*h*_ of 0.81 and 0.75, and the stress subscale had a *ω*_*t*_ and *ω*_*h*_ of 0.85 and 0.79.

The English DASS-Y was translated into Chinese adopting forward-backward translating method in consideration of both linguistic and cross-cultural equivalence [59]. The translation procedures followed are listed below.

The original English version (V1) of the DASS-Y was separately sent to two bilingual translators whose native language was Chinese to be translated into Chinese versions (V2 & V3 forward translation). After translation, the corresponding author discussed any inconsistencies with the two translators to generate a mutually acceptable Chinese version (V4). Then V4 was sent to a Chinese language teacher for expression optimization (V5). Following this, the corresponding author contacted a third bilingual translator to conduct back-translation to get another English version of the DASS-Y (V6). Following this, the corresponding author convened an expert panel consisting of three translators, a Chinese language teacher, and expertise in on pediatrics, public health, and psychometrics to review all six versions of the DASS-Y. Through comparison and discussion, with a view to being loyal to the original meaning and adaptation to the Chinese culture, the seventh Chinese version (V7) of the DASS-Y was approved by the expert panel. Following this, a small-scale pilot study was conducted in which six primary and middle school students were invited to complete the scale, with the purpose of checking whether they could comprehend the translated items and complete the scale on their own. Through interviews with these students, it was found that the language of the scale was easy to read, and that the content could be clearly understood. Based on the aforementioned procedures, the final version (V7) of the DASS-Y (see Table S1) was developed and approved.

It needs to be noted that because the DASS-Y is developed for youth, it comprises easier wording, as well as simple and short sentences. This reduced the difficulty of translation avoiding violation of grammatical–syntactical, and idiomatic equivalence [60]. However, issues of conceptual and experiential equivalence were still encountered as found in the previous study during the translation process [60]. For example, in terms of vocabulary or conceptual equivalence, the English word *“hate”* can be used to express *“strong hostility or dissatisfaction”* or *“dislike” *in Chinese. In this case, the translation team members in the present study engaged in thorough discussion and detailed evaluation of Chinese words, selecting those most appropriate for the developmental level/life stage/mental state of youth. *“My heart beating” *can be due to different reasons, such as an individual meeting someone they love or doing exercise. When translating, appropriate background knowledge needs to be supplemented.

#### Maslach Burnout Inventory–Student Survey (MBI-SS)

Emotional exhaustion was assessed in this study using the five-item subscale of the Maslach Burnout Inventory–Student Survey (MBI-SS) [61]. All items are rated on a seven-point Likert scale ranging from zero (*never*) to six (*always*), in order to assess the degree to which participants experience academic emotional exhaustion as described in each prompt. An example item is *“I feel used up at the end of a school day”* [62]. The exhaustion subscale proved to have excellent internal reliability with the McDonald’s ω value being 0.90 for middle school students and 0.94 for primary school students.

### Data analysis

The analytical approach employed in the present study involved using Rasch analysis to evaluate each item in the DASS-21 and DASS-Y, particularly focusing on item validity. This was followed by the application of CFA to assess the factorial, convergent, and discriminant validity. Subsequently, the concurrent validity of the two scales was rigorously evaluated in relation to emotional exhaustion. It is worth noting that previous research has established a substantial association between emotional exhaustion and students’ emotional disorders [53, 54]. For the purpose of the present study, structural equation modelling (SEM) was employed as the primary statistical analysis method. Detailed procedures relating to these methods are elaborated upon below.

Initially, descriptive statistics were computed for primary and middle school students, encompassing the means and standard deviations of DASS-21, DASS-Y, their respective subscales, and emotional exhaustion. In concordance with the cutoff values provided by the developers of DASS-21 [63] and DASS-Y [64], the proportion of participants who exhibited clinically significant emotional symptoms as indicated by both scales was calculated. Following this, Pearson correlation analysis was conducted to scrutinize the relationships among these variables.

In order to evaluate item validity, Rasch analysis was carried out following the guidelines proposed by Tennant and Conaghan [65]. The analysis involved selecting a suitable model (either the Partial Credit Rasch Model or the Andrich Rating Scale Model), and considering the following aspects: (i) assessing unidimensionality by the eigenvalue ratio criterion [66], (ii) verifying category ordering through a person-item map, and (iii) analyzing item validity utilizing information-weighted fit statistics (INFIT) and outlier-sensitive fit statistics (OUTFIT). Acceptable ranges for valid item responses were established between 0.50 and 1.50 [67]. Furthermore, scale targeting, differential item functioning (DIF), and person separation reliability were evaluated. Scale targeting was scrutinized using the Wright map, DIF was assessed through item difficulty differences between groups [68], and person separation reliability was established at 0.50 following the suggestion of Boone et al. [68].

Upon completion of the Rasch analysis, to examine the construct validity of the scale, CFA was implemented. Given the ordinal nature of the response options, the Diagonally Weighted Least Squares (DWLS) estimation method was specifically employed in the analytical process [69]. The criterion for factorial validity followed the standard proposed by Hu and Bentler [70], which stipulates that for a good fit, the comparative fit index (CFI) and the non-normed fit index (NNFI) should exceed 0.90, while the root mean square error of approximation (RMSEA) and standardized root mean square residual (SRMR) should fall below 0.06 and 0.08, respectively. In addition, the Akaike information criterion (AIC) was used to compare the model fit of the modified scale post-item diagnosis through Rasch analysis with that of the original scale [71]. Convergent and discriminant validity were evaluated using composite reliability (CR) and average variance extracted (AVE), following the guidelines outlined by Hair et al. [72]. More specifically, a CR greater than 0.70 and an AVE greater than 0.50 were deemed indicative of convergent validity for each construct. Discriminant validity was assessed by verifying that the square root of the AVE of the construct exceeded its correlation with other latent variables.

In the final stage of the analysis, SEM was utilized to explore the relationship between the three subscales of the DASS-21 and the DASS-Y (tested separately) with emotional exhaustion, which was assessed using the widely recognized MBI-SS. As aforementioned, emotional exhaustion is a construct empirically associated with psychological distress among primary and middle school students [53, 54], and the MBI-SS has been validated as a scale with superior psychometric attributes [73]. SEM was adopted for two main reasons. First, it allowed for the examination of the individual influence of three emotional disorders—depression, anxiety, and stress—on emotional exhaustion while controlling for the association of the other two disorders. Second, SEM facilitated the integration of gender as a control variable and allowed for the mitigation of measurement errors. Therefore, this methodology provides a more accurate elucidation of concurrent validity, an advantage unattainable through zero-correlation. Consequently, separate SEM models were constructed for primary and middle school students, with gender incorporated as a control variable, to examine the relationship between depression, anxiety, stress, and emotional exhaustion.

## Results

### Examining the mean differences and Pearson correlations between DASS-21 and DASS-Y

Table 1 shows the means (SDs) and Pearson correlation outcomes for the DASS-21 and DASS-Y in the two samples (i.e., primary and middle school students). For primary school students, a small effect size discrepancy was found in the anxiety subscale between the DASS-21 and DASS-Y (*t* = 8.71, *p* < 0.01, Cohen’s *d* = 0.22). This discrepancy signified a lower score on the DASS-Y compared to the DASS-21. However, despite achieving statistical significance, the effect sizes for the depression and stress subscales demonstrated negligible differences between the DASS-21 and DASS-Y (Depression: *t*=-3.98, *p* < 0.01, Cohen’s *d* = 0.10; Stress: *t*=-5.42, Cohen’s *d* = 0.14). The proportions of individuals displaying clinical-level emotional symptoms, as classified by the two scales, exhibited significant heterogeneity. For example, using the DASS-Y, the proportion of students identified as depressed was 9.6% for primary school students and 21.3% for middle school students. The proportions of students identified as depressed using the DASS-21 were 14.5% for primary school students and 44.7% middle school students (see Table S2). Moreover, the DASS-Y had a significant reduction in the proportion of individuals identified with clinical-level emotional symptoms in comparison to the DASS-21. This was corroborated by the McNemar-Bowker tests, with the chi-square values reaching statistical significance (*χ*^2^ values ranged from 52.60 to 81.16, all satisfying *p* < 0.01).

**Table 1:** Mean and the correlation among the observed variables

Primary school students (*N* = 1507)										
	*Mean (SD)*	1	2	3	4	5	6	7	8	9
1. DASS-21	5.49 (8.62)	1.00								
2. DASS-Y	5.62 (7.77)	0.64	1.00							
3. DASS-21 Depression	1.62 (2.99)	0.96	0.60	1.00						
4. DASS-Y Depression	1.92 (2.97)	0.55	0.90	0.53	1.00					
5. DASS-21 Anxiety	1.78 (2.94)	0.96	0.62	0.88	0.51	1.00				
6. DASS-Y Anxiety	1.22 (2.49)	0.58	0.89	0.55	0.74	0.58	1.00			
7. DASS-21 Stress	2.09 (3.07)	0.96	0.63	0.86	0.53	0.87	0.55	1.00		
8. DASS-Y Stress	2.49 (3.23)	0.58	0.89	0.52	0.67	0.56	0.68	0.59	1.00	
9. Emotional exhaustion	2.65 (3.93)	0.63	0.52	0.61	0.47	0.59	0.43	0.61	0.48	1.00
Middle school students (*N* = 1131)										
	*Mean (SD)*	1	2	3	4	5	6	7	8	9
1. DASS-21	14.88 (11.72)	1.00								
2. DASS-Y	12.86 (9.16)	0.72	1.00							
3. DASS-21 Depression	4.70 (4.29)	0.93	0.68	1.00						
4. DASS-Y Depression	4.05 (3.47)	0.60	0.86	0.63	1.00					
5. DASS-21 Anxiety	4.83 (4.09)	0.94	0.65	0.80	0.52	1.00				
6. DASS-Y Anxiety	2.94 (3.07)	0.62	0.83	0.55	0.62	0.63	1.00			
7. DASS-21 Stress	5.36 (4.16)	0.94	0.67	0.80	0.54	0.83	0.57	1.00		
8. DASS-Y Stress	5.87 (4.11)	0.62	0.88	0.57	0.61	0.55	0.58	0.62	1.00	
9. Emotional exhaustion	6.49 (4.63)	0.51	0.46	0.51	0.39	0.45	0.33	0.47	0.44	1.00

All *p* < 0.01

The results for middle school students mirrored that of primary school students. The DASS-Y anxiety subscale scores were significantly lower than those on the DASS-21, yielding a small effect size (*t* = 19.79, *p* < 0.01, Cohen’s *d* = 0.29). The raw scores on the depression and stress subscales followed a similar trend. However, despite statistically significant differences, the effects were trivial (depression: *t* = 6.39, *p* < 0.01, Cohen’s *d* = 0.19; Stress: *t*=-4.82, Cohen’s *d* = 0.14). With regard the diagnosis of clinical mood symptoms, the proportion of middle school students identified with clinically significant manifestations using the DASS-Y was significantly lower compared to those diagnosed using the DASS-21 (*χ*^2^ values ranged from 177.78 to 531.07, all satisfying *p* < 0.01) (see Table S2). This pattern echoed the findings in the primary school cohort.

In terms of all variables, namely the whole scale, three subscales, and emotional exhaustion, significant positive correlations were observed (primary school students: *r* between 0.51 and 0.96; middle school students: *r* between 0.39 and 0.94). Of note, the correlation coefficients between depression, anxiety, and stress were reduced in the DASS-Y compared to the DASS-21, decreasing from coefficients of over 0.80 to between 0.50 and 0.74, which underscores a refinement in the overlap among the three mood disorders.

### Rasch analysis

As a result of the significant likelihood ratio test (with *χ*^*2*^ ranging from 21.49 to 144.82, all *p* < 0.05) in both the DASS-21 and DASS-Y across primary school students and middle school students, the Partial Credit Model (PCM) was used rather than the Rating Scale Model (RSM). The Rasch analysis findings indicated that all three subscales in both the DASS-21 and DASS-Y satisfied the unidimensionality criterion, as shown by the eigenvalue ratios ranging from 3.27 to 8.40. The majority of these ratios surpassed 4, thereby reinforcing the unidimensionality of the scales. Nevertheless, within the primary school students, a number of items exhibited disordered categories in both the DASS-21 and DASS-Y. For the DASS-21, these included Items 5, 13, and 16 in the depression subscale; Items 8, 9, 10, 11, 13, and 14 in the anxiety subscale; and Items 1, 6, 8, 12, 14, and 18 in the stress subscale. As for the DASS-Y, Items 2 to 5 in the depression subscale, Items 8 to 11, 13, and 14 in the anxiety subscale, and all items in the stress subscale displayed category disordering. In contrast, the middle school students exhibited a reduced incidence of category disordering, with only Item 20 in the anxiety subscale of the DASS-21 showing such disordering. Similarly, in the DASS-Y, only Items 4 and 6 in the depression subscale, and Items 10 and 13 in the anxiety subscale exhibited this characteristic.

To rectify this issue, a common strategy of collapsing categories was adopted. Following the amalgamation of responses (by unifying categories 2 and 3 into a single category for items that required correction), a systematic augmentation was observed in the category calibration of all items. Post-correction, item diagnostics was conducted on the adjusted items, and the outcomes indicated that the INFIT and OUTFIT of all items were within the acceptable range (0.50 to 1.50) on both the DASS-21 and DASS-Y among primary and middle school students (see Tables 2 and 3).

**Table 2:** Psychometric properties of DASS-21 in item level on primary and middle school students

	Primary school students				Middle school students				DIF contrast
	Difficulty	Point-biserial	INFIT MnSq	OUTFIT MnSq	Difficulty	Point-biserial	INFIT MnSq	OUTFIT MnSq	
Subscale of Depression (person separation reliability = 0.70 and 0.71)									
3. Unable to experience positive emotions	0.04	0.78	1.25	1.23	0.01	0.73	0.93	0.94	0.03
5. Lack of initiative	-0.76	0.78	1.20	1.21	-0.15	0.66	1.29	1.29	0.61
10. Absence of anticipation for the future	0.15	0.78	1.04	1.02	-0.26	0.70	1.17	1.15	0.41
13. Feeling sad or depressed	-0.33	0.81	0.87	0.88	-0.12	0.75	0.91	0.91	0.21
16. Difficulty feeling enthusiastic	-0.52	0.79	1.01	1.02	0.20	0.71	0.98	0.94	0.72
17. Low self-worth	0.74	0.79	0.73	0.62	0.09	0.72	0.85	0.80	0.65
21. Life feels meaningless	0.67	0.75	0.89	0.83	0.23	0.69	0.92	0.94	0.44
Subscale of Anxiety (person separation reliability = 0.65 and 0.68)									
2. Dry mouth	0.07	0.74	1.31	1.29	-0.03	0.69	1.02	1.02	0.10
4. Difficulty breathing	0.05	0.72	0.94	0.93	0.72	0.64	0.90	0.81	0.67
7. Trembling	0.20	0.74	0.86	0.77	0.58	0.64	1.03	0.97	0.38
9. Fear of embarrassing oneself in panic situations	-0.65	0.79	1.19	1.29	-1.60	0.68	1.41	1.38	0.95
15. Feeling close to panic	-0.17	0.75	0.87	0.84	0.16	0.68	0.93	0.89	0.33
19. Awareness of heart palpitations	0.12	0.76	0.69	0.63	0.32	0.68	0.88	0.86	0.20
20. Irrational fear	0.38	0.75	0.97	0.93	-0.14	0.67	0.82	0.79	0.52
Subscale of Stress (person separation reliability = 0.66 and 0.72)									
1. Difficulty winding down and relaxing	-0.56	0.74	1.24	1.23	-0.06	0.66	1.12	1.13	0.50
6. Over-reactivity and excessive use of nervous energy	0.12	0.72	1.04	1.03	0.41	0.63	1.03	1.04	0.29
8. Agitation and getting easily agitated	0.00	0.78	0.85	0.83	-0.25	0.73	0.94	0.92	0.25
11. Intolerance and being touchy	0.95	0.76	0.82	0.76	0.09	0.72	0.85	0.82	0.86
12. Difficulty relaxing and unwinding	0.07	0.78	0.73	0.67	-0.01	0.73	0.77	0.73	0.08
14. Tendency to overreact to situations	-0.25	0.73	1.17	1.18	-0.05	0.63	1.24	1.25	0.20
18. Feeling touchy	-0.35	0.74	1.15	1.15	-0.14	0.67	1.05	1.11	0.21

**Table 3:** Psychometric properties of DASS-Y in item level on primary and middle school students

	Primary school students				Middle school students				DIF contrast
	Difficulty	Point-biserial	Infit MnSq	Outfit MnSq	Difficulty	Point-biserial	Infit MnSq	Outfit MnSq	
Subscale of Depression (person separation reliability = 0.62 and 0.68)									
1. I hated my life	-0.39	0.74	1.36	1.39	0.14	0.72	0.97	0.96	0.53
2. I hated myself	-0.06	0.74	0.85	0.85	0.54	0.69	0.93	0.87	0.60
3. I felt that life was terrible.	-0.29	0.78	0.81	0.81	0.03	0.75	0.86	0.85	0.32
4. I felt like I was no good	-0.21	0.76	0.82	0.85	-1.01	0.72	0.94	0.93	0.80
5. I could not stop feeling sad	-0.16	0.75	0.92	0.91	0.23	0.70	0.97	0.99	0.39
6. I did not enjoy anything	0.97	0.71	0.95	0.98	0.17	0.59	1.08	1.21	0.80
7. There was nothing nice I could look forward to.	0.12	0.69	1.29	1.38	-0.09	0.63	1.26	1.33	0.21
Subscale of Anxiety (person separation reliability = 0.62 and 0.54)									
8. I felt like I was about to panic	-0.20	0.77	1.01	0.99	-0.32	0.71	0.97	0.95	0.12
9. I felt terrified	-0.49	0.80	0.92	0.88	-0.12	0.71	0.89	0.85	0.37
10. I felt scared for no good reason	-0.69	0.78	1.09	1.09	-0.36	0.63	0.95	0.93	0.33
11. I could feel my heart beating	-0.29	0.77	1.02	1.06	-0.04	0.67	1.06	1.04	0.25
12. I had trouble breathing	1.19	0.69	0.99	1.02	0.68	0.58	1.03	1.02	0.51
13. My hands felt shaky	0.05	0.73	1.00	1.02	-0.59	0.64	1.04	1.03	0.64
14. I felt dizzy, like I was about to faint	0.44	0.71	0.92	1.03	0.74	0.57	1.08	1.19	0.30
Subscale of Stress (person separation reliability = 0.71 and 0.75)									
15. I got upset about little things	-0.16	0.79	1.02	1.01	-0.53	0.73	0.96	0.95	0.37
16. I was easily irritated	-0.63	0.79	1.06	1.05	0.11	0.68	1.01	0.98	0.74
17. I found myself over-reacting to situations	0.20	0.76	0.99	0.97	0.24	0.68	0.96	0.98	0.04
18. I was easily annoyed	0.08	0.82	0.76	0.72	-0.23	0.75	0.82	0.81	0.31
19. I was stressing about lots of things	0.27	0.78	0.93	0.89	-0.09	0.73	0.88	0.88	0.36
20. I got annoyed when people interrupted	-0.45	0.74	1.28	1.30	0.05	0.63	1.21	1.26	0.50
21. I found it difficult to relax	0.69	0.72	0.94	0.88	0.47	0.60	1.17	1.23	0.22

**Fig. 1:**
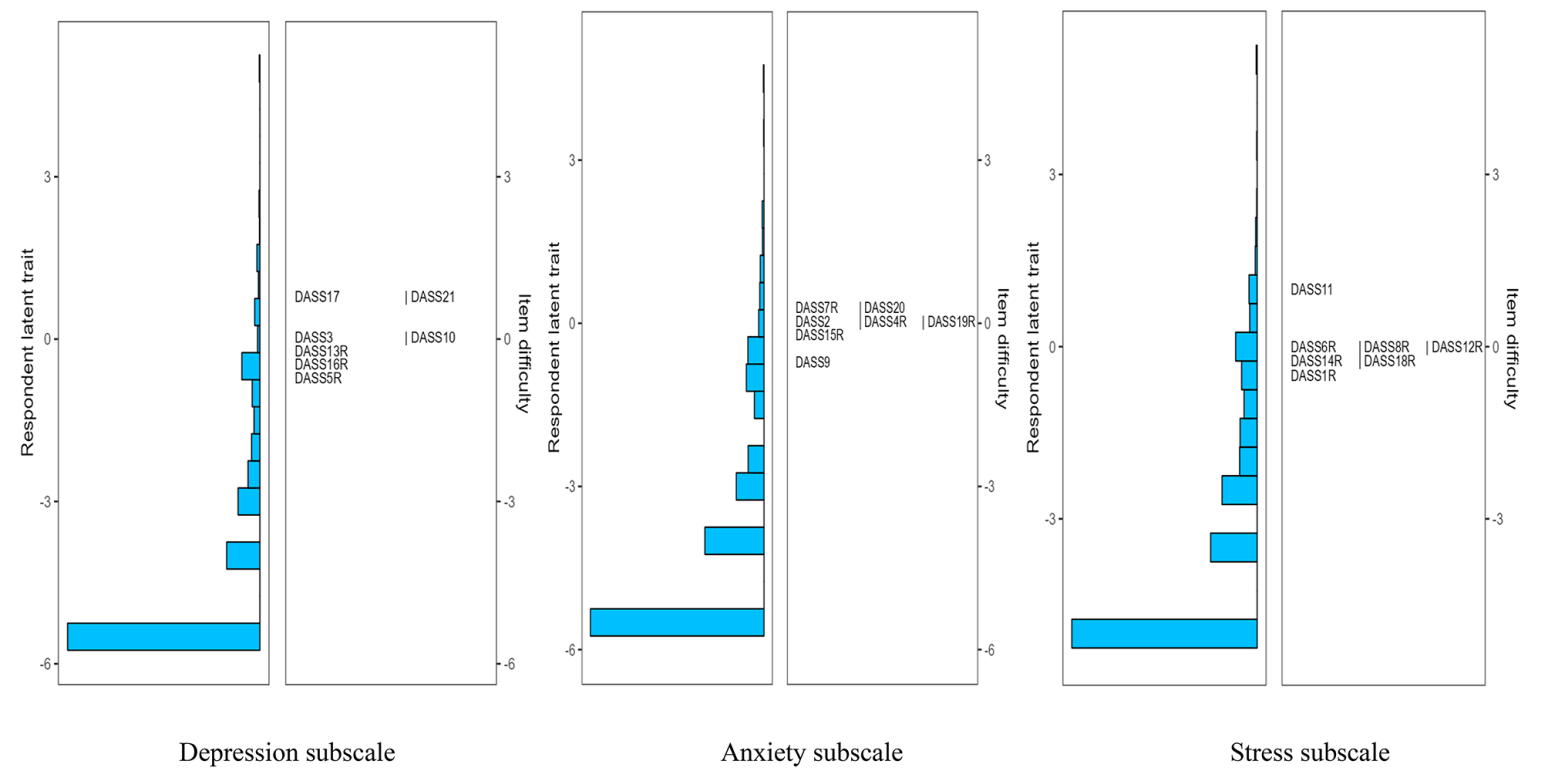
DASS-21 among primary school students

**Fig. 2:**
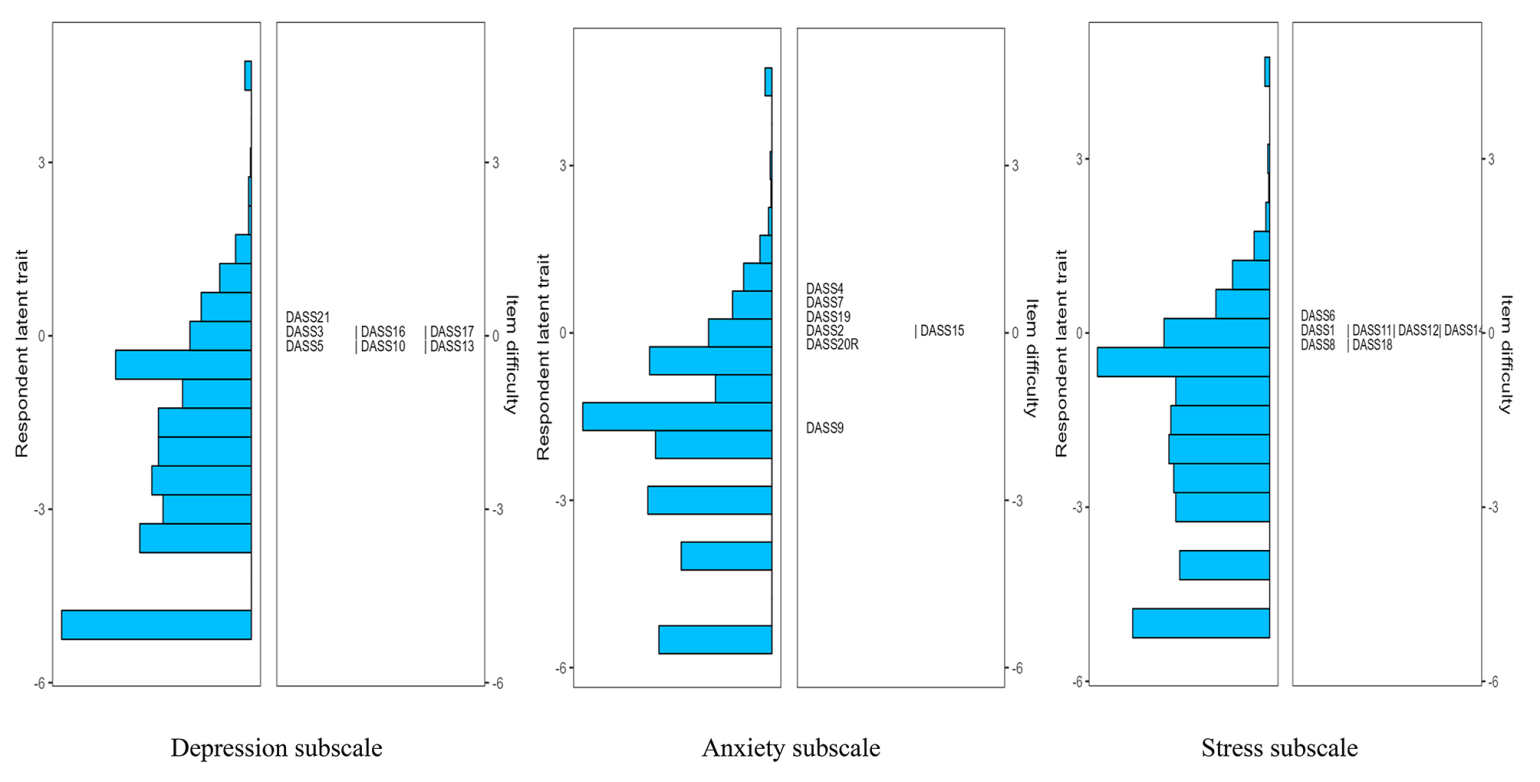
DASS-21 among middle school students

**Fig. 3:**
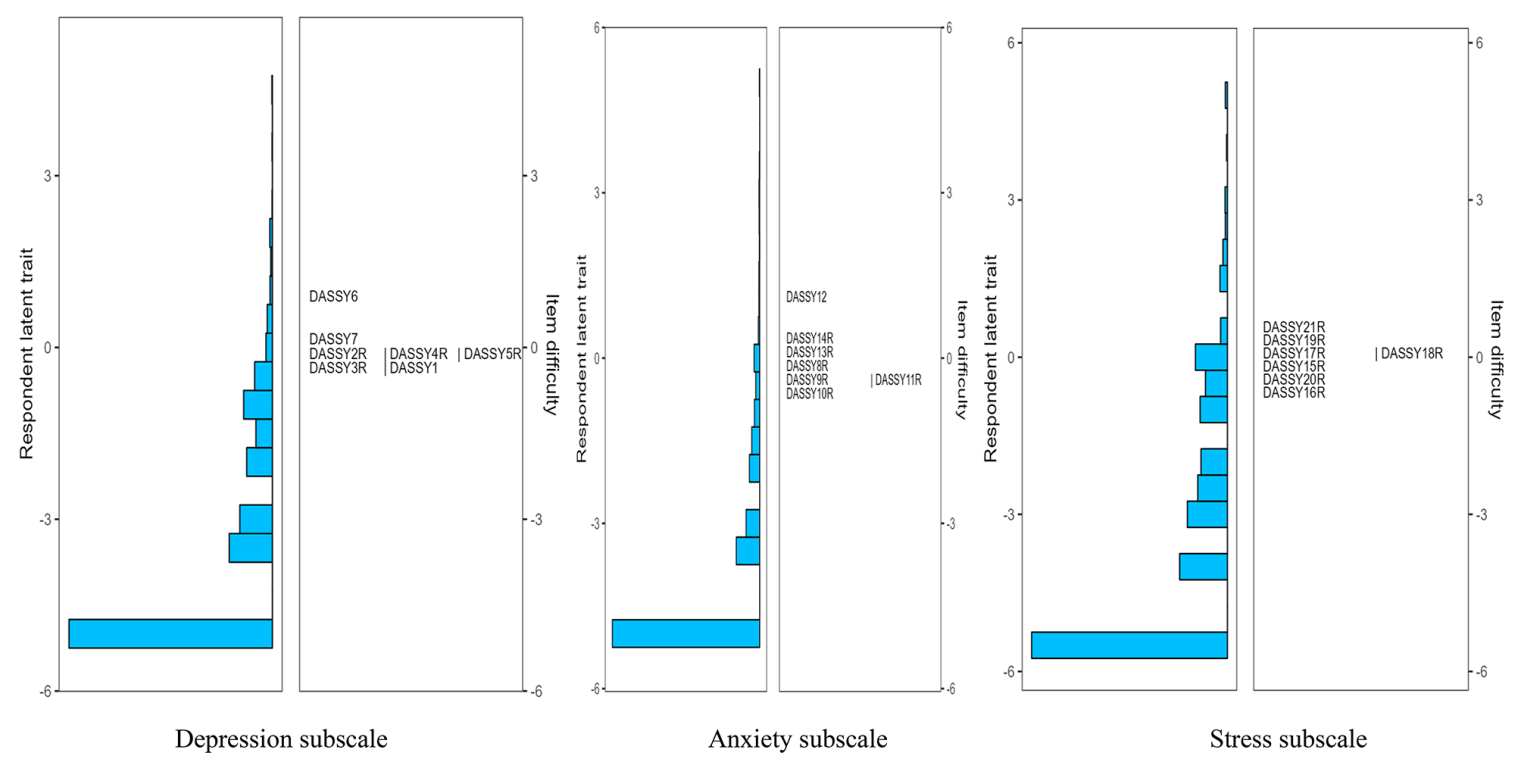
DASS-Y among primary school students

**Fig. 4:**
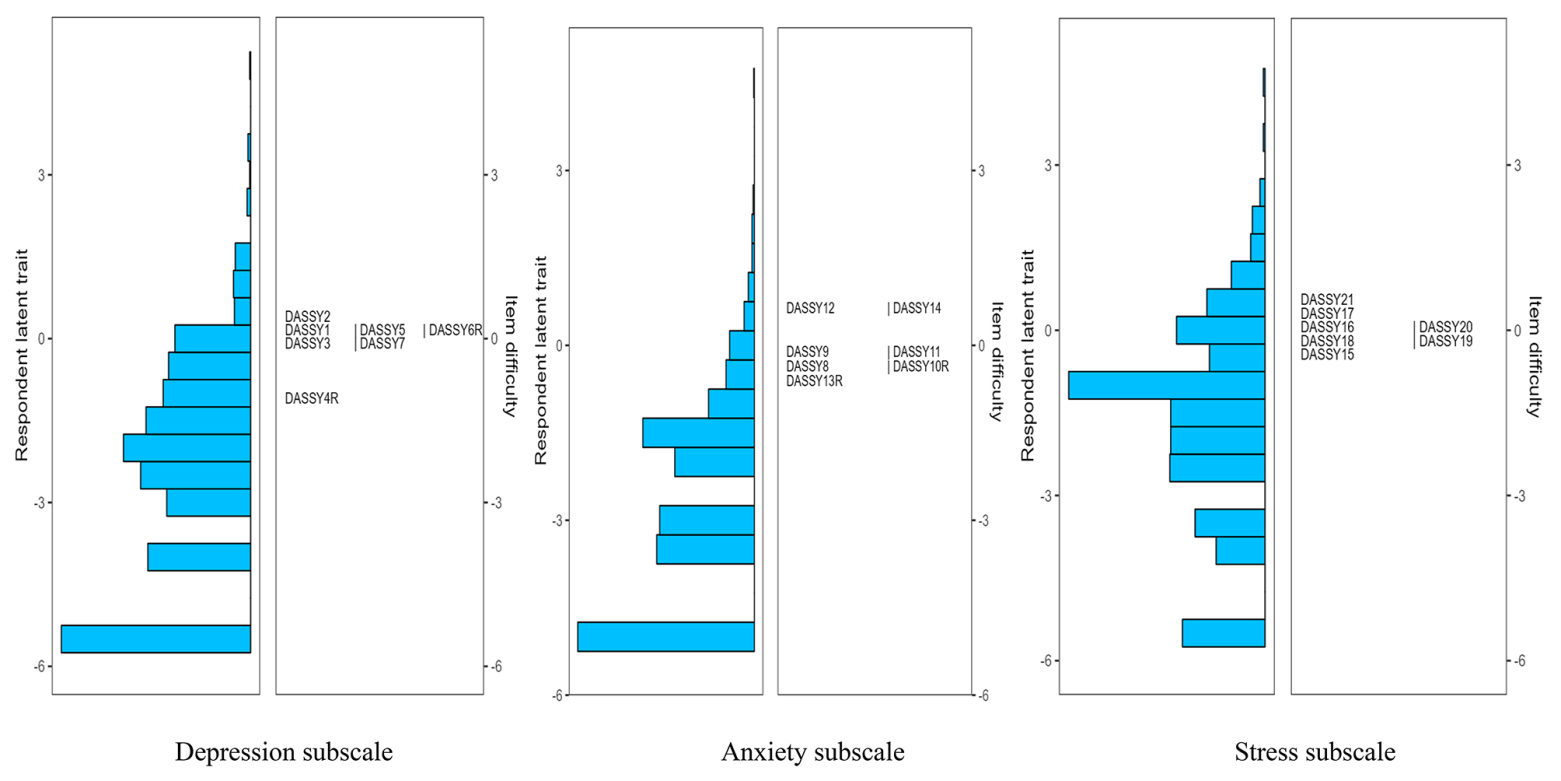
DASS-Y among middle school students

In terms of scale targeting, as shown by the results from the Wright map (see Figs. 1, 2, 3 and 4), the majority of primary school students possessed relatively low levels of traits in the three subscales of both DASS-21 and DASS-Y, with mean person measures spanning from − 3.27 to -4.09. A similar scenario was observed among middle school students, albeit the mean person measure was in closer proximity to the mean item measures, ranging from − 1.64 to -2.76. Furthermore, through the figures, the distribution of item difficulties suggested a significant improvement in the DASS-Y for children. In the original DASS-21 version, each subscale featured multiple items assessing traits at analogous levels. However, this redundancy was substantially ameliorated in the DASS-Y. Conversely, in terms of item distribution, the DASS-Y did not show significant improvement compared to the DASS-21 for middle school students.

A few items in the DASS-21 and DASS-Y had significant DIF between primary and middle school students. More specifically, in the DASS-21, Items 16 and 17 from the depression subscale, Items 4 and 9 from the anxiety subscale, and Item 11 from the stress subscale had DIF contrasts greater than 0.64. Similarly, in the DASS-Y, Items 4 and 6 from the depression subscale, and Item 16 from the stress subscale had significant DIF between the two groups.

Tables 2 and 3 outline the person separation reliability for the two revised scales post-category collapsed correction. Aside from a value of 0.54 for the anxiety subscale of the DASS-Y among middle school students, the person separation reliability for the remaining scales exceeded 0.60, thereby meeting the predetermined criteria.

### Confirmatory factor analysis

Table 4 delineates the comparative fit of the two scales within the framework of three unique factor structures. To provide further insight into the effect of category collapsing following Rasch item diagnosis, a version of model fit absent of any corrections is also presented (i.e., a comparison is made between fit results of the post-category collapsed correction and those of the original item category). The explication of the CFA results follows a sequenced approach.

The analysis was initiated by focusing on the model fit of DASS-21 and DASS-Y across the three distinct factor structures. For primary school students, irrespective of the implementation of Rasch’s category collapsing correction, neither DASS-21 nor DASS-Y resulted in an excellent model fit within any of the three factor structures. Instead, both the DASS-21 and DASS-Y showed adequate model fit for the three-factor structure. This pattern mirrors that observed among middle school students. In summary, among the three factor structures examined, the three-factor model emerged as the most suitable for both the DASS-21 and DASS-Y. This is evidenced by its lowest AIC value and the fact that its fit indices—CFI, NNFI, and SRMR—all conformed to the standard criteria. The only exception was the RMSEA value, which marginally surpassed the standard benchmark of 0.60.

Subsequently, the examination involved a comparison between the model fit of the original and the corrected items (via Rasch’s category collapsing). Findings suggested that the corrected model fit outperformed its uncorrected counterpart, as evidenced by a comparatively lower AIC.

Lastly, considering that the three-factor model yielded the lowest AIC, the Composite Reliability (CR) and Average Variance Extracted (AVE) were computed for the scales based on this factor structure’s loading (see Figures S1 and S2). As Table 5 shows, the DASS-21 and DASS-Y exhibited robust convergent validity in the primary school student sample, with CR values surpassing 0.80 and AVE values exceeding 0.50. Among middle school students, convergent validity was partially substantiated, with CR values remaining above 0.80, but with AVE values being somewhat diminished, ranging from 0.37 to 0.49. However, due to exceedingly high correlations among the three factors (all over 0.90), the discriminant validity of the DASS-21 was unsatisfactory in both samples. In the DASS-Y, the inter-factor correlations were lower for both samples, with the majority of coefficients below 0.80. However, the ideal standard for discriminant validity remained unmet.

**Table 4:** Model fit among DASS-21 and DASS-Y

Primary school students						
	**χ** ^**2**^ **(** ***df*** **)**	**CFI**	**NNFI**	**RMSEA**	**SSRMR**	**AIC**
One factor- DASS-21	1673.42 (189) 1735.91 (189)	0.931 0.929	0.923 0.921	0.072 0.074	0.032 0.033	32120.22 28328.81
One factor- DASS-Y	3153.86 (189) 2960.03 (189)	0.833 0.844	0.815 0.826	0.102 0.099	0.059 0.057	35268.55 30736.57
Two factor- DASS-21	1633.01 (188) 1694.01 (188)	0.933 0.931	0.925 0.923	0.071 0.073	0.032 0.032	32081.81 28288.91
Two factor- DASS-Y	2568.32 (188) 2371.27 (188)	0.866 0.877	0.851 0.862	0.092 0.088	0.054 0.052	34685.01 30149.82
Three factor- DASS-21	1610.05 (186) 1662.33 (186)	0.934 0.932	0.925 0.923	0.071 0.073	0.032 0.032	32062.86 28261.23
Three factor- DASS-Y	1473.45 (186) 1325.29 (186)	0.928 0.936	0.918 0.927	0.068 0.064	0.038 0.037	33594.14 29107.84
**Middle school students**						
	**χ** ^**2**^ **(** ***df*** **)**	**CFI**	**NNFI**	**RMSEA**	**SRMR**	**AIC**
One factor- DASS-21	1074.88 (189) 1059.75 (189)	0.923 0.924	0.914 0.915	0.064 0.064	0.036 0.036	47598.45 47276.23
One factor- DASS-Y	1795.49 (189) 1773.26 (189)	0.815 0.816	0.795 0.795	0.087 0.086	0.059 0.058	44328.06 43480.05
Two factor- DASS-21	1006.25 (188) 995.54 (188)	0.929 0.929	0.920 0.921	0.062 0.062	0.035 0.035	47531.81 47214.02
Two factor- DASS-Y	1378.51 (188) 1356.73 (188)	0.863 0.864	0.847 0.848	0.075 0.074	0.052 0.052	43913.07 43065.53
Three factor- DASS-21	982.05 (186) 972.75 (186)	0.931 0.931	0.922 0.922	0.062 0.061	0.035 0.035	47511.61 47195.23
Three factor - DASS-Y	824.84 (186) 818.80 (186)	0.927 0.926	0.917 0.917	0.055 0.055	0.038 0.038	43363.41 42531.59

CFI = comparative fit index; NNFI = non-normed fit index; RMSEA = root mean square error of approximation; SRMR = standardized root mean square residual; data in the line above is uncorrected, and in the line below is corrected after item diagnosis.

**Table 5:** Test of discriminant validity among the subscales of the DASS-21

Primary school students						
	DASS-21			DASS-Y		
	Depression	Anxiety	Stress	Depression	Anxiety	Stress
Depression	**0.77**			**0.71**		
Anxiety	0.98	**0.73**		0.86	**0.72**	
Stress	0.97	0.97	**0.72**	0.77	0.77	**0.74**
Composite reliability	0.91	0.89	0.88	0.88	0.88	0.89
**Middle school students**						
	DASS-21			DASS-Y		
	Depression	Anxiety	Stress	Depression	Anxiety	Stress
Depression	**0.70**			**0.65**		
Anxiety	0.95	**0.66**		0.75	**0.61**	
Stress	0.94	0.96	**0.66**	0.73	0.70	**0.66**
Composite reliability	0.87	0.84	0.84	0.84	0.80	0.84

*Notes*: Diagonal elements in bold are square root of averaged variance extracted. When these values were higher than the inter-latent factors correlations (off-diagonal elements), the discriminant validity was support for the respective latent variable. CR of depression, anxiety, and stress was in DASS-Y among primary school students

### Concurrent validity

Finally, SEM was used to further examine the relationship between DASS-21, DASS-Y, and academic emotional exhaustion among both primary and middle school students, to assess the concurrent validity of these scales. Notably, the indicators used in the analysis was refined through a process known as category collapsing. With gender as a control variable, the SEM results demonstrated that DASS-21 provided a satisfactory model fit for both primary and middle school students, as evidenced by the following fit indices: *χ*^2^ (315) = 2483.92 and 2703.93, RMSEA = 0.068 and 0.082, CFI = 0.985 and 0.974, NNFI = 0.983 and 0.971, SRMR = 0.033 and 0.044. Nevertheless, the path coefficients indicated a few unanticipated associations between the three subscales and emotional exhaustion, particularly concerning the relationship between anxiety and emotional exhaustion, despite the presence of significant positive correlations between depression, stress, and emotional exhaustion for both student groups (see Figure S3). More specifically, both primary and middle school students exhibited a pronounced negative correlation between anxiety and emotional exhaustion, with values of -0.61 and − 0.76, respectively, with the latter reaching a significance level of *p* = 0.004 in the middle school sample. Conversely, given that the DASS-Y presented a satisfactory model fit for both primary and middle school students (the model fit indices for primary and middle school students are as follows: *χ*^2^ (315) = 2032.65 and 2602.08, RMSEA = 0.060 and 0.080, CFI = 0.983 and 0.964, NNFI = 0.981 and 0.960, SRMR = 0.035 and 0.047), the three subscales resulted in a more rational correlation with emotional exhaustion. That is, depression and stress still exhibited a significant positive correlation with emotional exhaustion, while the correlation between anxiety and emotional exhaustion was virtually negligible (see Figure S4).

## Discussion

Considering the fact that mental health status of children and adolescents is deteriorating [10], a reliable and valid instrument used to assess psychological distress among this population is urgently needed. In view of the fact that the DASS-21, originally designed for adults, has courted controversy when used among children and adolescents [16, 41], the DASS-Y, a newly developed version of DASS was specifically developed for this cohort. However, the DASS-Y has not been validated in other cultures and populations. Therefore, the present study translated the English DASS-Y into simplified Chinese and then evaluated and compared the psychometric properties of these two scales with a sample of Chinese primary and middle school students.

The results indicated that there were significant differences between the DASS-21, originally developed for adults, and the DASS-Y, specifically designed for children and adolescents, when tested among young students. A non-negligible effect size was observed in the anxiety subscale between the two scales. In addition, there were significant discrepancies in the proportion of individuals classified as having clinical mood disorder according to the cutoff value of the two scales. Moreover, a Rasch analysis was conducted, demonstrating that several items in both scales had disordered categories among primary school students. Interestingly, items with disordered categories were only sporadic among middle school students. After introducing a collapsing response, both scales exhibited ideal internal reliability, factorial validity, and convergent validity. However, when considering the distribution of item difficulties, discriminant validity, and concurrent validity, the DASS-Y is deemed to be more appropriate for use with children and adolescents compared to the DASS-21. Furthermore, fewer items in the DASS-Y demonstrated DIF than those in the DASS-21. A more detailed discussion of this now follows.

First, regarding to the prevalence of mood disorders between two scales, it is found that the percentage of students diagnosed with mental health problems in the DASS-Y was much lower than that in the DASS-21, either from primary school or middle school. The outcomes derived from the DASS-Y, which classed 9.6% of primary school students and 21.3% of middle school students as depressed, align closely with the report from the National Mental Health Development in China (2021–2022). This report by the Chinese Academy of Science (CAS) in 2023 and authored by Guo et al. [74], used the Center for Epidemiological Studies Depression Scale (CES-D) with a nationally representative sample. Notably, this comprehensive study involved the participation of over 30,000 primary and middle school students in an online survey. The study reported that 10.8% of primary school students and 19% of middle school students were depressed. In comparison, the values of primary school students and middle school students obtained by DASS-21 in the present study were 14.5% and 44.7% respectively, which far exceed the values in the CAS study. Considering that the CAS study used a representative sample, the results obtained from the DASS-Y in the present study are closer to the current mental health status of primary and middle school students in China. The DASS-21 was originally developed for adults and based on the findings of the present study, overestimated the number of students with psychological distress. This finding supports previous studies which have found that depression among children and adolescents cannot be accurately estimated using an adult depression scale [41] and which have warned against using adult measures among adolescent populations [16].

Second, the Rasch analysis demonstrated item validity concerns within both the DASS-Y and DASS-21, with disordered categories being particularly prevalent in the responses from primary school students. This may have arisen due to the 0–3 scoring scheme, where a majority of the primary school participants leaned towards the ‘2’ category, indicating a lack of discernment between scoring categories 2 and 3. While the present study managed to enhance the overall psychometric quality of the scale by adjusting the scoring method, it necessitated altering the cutoff points used to determine the clinical prevalence of mood disorders. A well-validated and meaningful cutoff score carries significant importance for the effective screening of psychological distress among adolescents [75]. Given that Szabó and Lovibond [11] did not employ Rasch analysis in the development of the DASS-Y, the present study represents a significant contribution to this field. By addressing and rectifying the issues relating to item validity and scoring within the scales, an improved tool for evaluating the emotional wellbeing of children and adolescents is provided here.

Third, the present study explored the factorial validity of the DASS-21 and DASS-Y, comparing one-factor, two-factor, and three-factor structures. The analysis showed that the DASS-Y only approached the set standard for model fit for the three-factor structure, as seen among both primary and middle school students. This validates the three-factor structure in the Chinese DASS-Y and matches the findings of the original English version [11]. The DASS-21 showed a near ideal model fit for all three-factor structures, but the three-factor structure was the best. The factorial validity of the DASS-21 has been debated in previous studies, with single-factor or bifactor models often showing ideal fit results [42]. This is inconsistent with the original authors’ view [15] that the three-factor structure is the best for the scale. For instance, Cao et al. [76] reported that a one-factor and three-factor structure for the DASS-21 were both acceptable, but that the latter was superior. Lee and Kim [47] proposed a bifactor structure, with a general factor and specific factors of depression, anxiety, and stress as the best fit model for the DASS-21. Jovanović et al. [42] suggested a bifactor-ESEM model as the optimal representation of the DASS-21 structure. Despite these differences regarding the DASS-21’s factor structure, the fact that the DASS-Y’s model fit was closest to the standard only in the three-factor structure and was a poor fit in one and two-factor structures for both primary and middle school students, emphasizes its factorial validity. These findings suggest that reducing the scale to a single factor or dividing it into two factors contradicts the theoretical underpinnings, compromising the scale’s intended structure. Moreover, given that the model fit of the DASS-Y within the three-factor structure did not entirely conform to the predetermined standards, there remains a significant need for ongoing validation studies on the factorial integrity of the Chinese version of DASS-Y in future research.

Fourth, the present study evaluated the convergent, discriminant, and concurrent validity of the DASS-21 and DASS-Y. The values of composite reliability (CR) and average variance extracted (AVE) demonstrated robust convergent validity for both scales among primary school students. This concurs with previous findings relating to the DASS-21 [75, 77], although this validity was only partially supported among middle school students. Contrary to previous studies that reported satisfactory discriminant validity for the DASS-21 [37–39], the present study’s findings indicated unsatisfactory levels among both primary and middle school students. However, the DASS-Y demonstrated an improvement in discriminant validity, albeit still falling short of ideal levels. This addressed the issue of high correlation among the depression, anxiety, and stress subscales, implying that these three subscales can be comparatively utilized to assess individual types of negative affect. Furthermore, the present study included academic emotional exhaustion as a factor to evaluate the concurrent validity of both scales post-refinement through category collapsing. The findings indicated that the DASS-Y exhibited superior concurrent validity compared to DASS-21, as shown by the path coefficients. This suggests the DASS-Y, in its revised form, may be a more effective instrument for assessing psychological distress among students compared to its predecessor.

## Limitations and directions for future research

The present study, while contributing valuable insights, does have a few limitations that may provide opportunities for future research. First, as a considerable number of items in the DASS-Y were identified as having disordered categories for primary school students through the Rasch analysis, it is recommended that the cutoff value is adjusted for this population. The repercussions of such a modification on the final results are uncertain and not within the scope of the present study’s predictions. Therefore, future research should concentrate on evaluating the outcomes after eliminating the issues of disordered category items and adjusting the cutoff value. Second, due to the use of convenience and purposive sampling for participant recruitment, the sample was predominantly drawn from primary school students in two southwestern Chinese cities and middle school students from central provincial capitals. This sampling strategy may create limitations in the generalizability of the findings to broader populations. Therefore, psychometric studies using more representative samples are needed in future. Third, although the Chinese version of DASS-Y exhibited satisfactory concurrent validity and strong internal reliability, it should be noted that the fit index for the factorial validity, the RMSEA, was marginally elevated. This contributed to the three-factor structure model not fully conforming to all established criteria. Considering that the DASS-Y is a new scale, the results of the present study do not conclusively eliminate the potential existence of alternate factor structures inherent to the scale. Consequently, future studies should further explore this issue. Fourth, in the translation process of the scale, a small pilot study was carried out to ascertain that potential participants could adequately comprehend the scale items. However, comprehensive qualitative analysis, including extensive interviews, was not conducted. Considering that the DASS-Y is a newly translated instrument targeted at youth, the clarity and accessibility of language expression is of pivotal importance. As such, additional rigorous qualitative investigation to further examine and ensure its comprehensibility is recommended. Fifth, with respect to criterion validity, the present cross-sectional study only evaluated the concurrent validity of the two scales. Future longitudinal research should also assess predictive validity. Such an examination would facilitate a better understanding of how well the scale score predicts future outcomes. Consequently, a higher predictive validity could enable more accurate decision-making.

## Conclusions

Despite the aforementioned limitations, being the first to translate the original English DASS-Y into another language, the present study demonstrated that the Chinese version of DASS-Y possessed similar psychometric properties to the English version. Consequently, the Chinese DASS-Y can be reliably, validly, and more appropriately applied to assess depression, anxiety, and stress among Chinese children and adolescents compared to the DASS-21.

### Electronic supplementary material

Below is the link to the electronic supplementary material.

Supplementary Material 1

Supplementary Material 2

## Data Availability

The datasets used and/or analyzed during the current study are available from the corresponding author on reasonable request.

## Abbreviations

DASS-Y: Depression Anxiety Stress Scale for Youth
DASS-21: Depression, Anxiety, and Stress Scale*–*21*-*item version
CFA: Confirmatory factor analysis
SEM: Structural equation modeling
MBI-SS: Maslach Burnout Inventory–Student Survey
INFIT: Information-weighted fit statistics
OUTFIT: Outlier-sensitive fit statistics
DIF: Differential item functioning
CFI: Comparative fit index
NNFI: Non-normed fit index
RMSEA: Root mean square error of approximation
SRMR: Standardized root mean square residual
AIC: Akaike information criterion
CR: Composite reliability
AVE: Average variance extracted
PCM: Partial Credit Model
RSM: Rating Scale Model
CAS: Chinese Academy of Science
CES-D: Center for Epidemiological Studies Depression Scale
