# Supplementary material for: Evaluating the psychometric properties of the Chinese Depression Anxiety Stress Scale for Youth (DASS-Y) and DASS-21
Source: Child Adolesc Psychiatry Ment Health. 2023 Sep 7;17:106. doi: 10.1186/s13034-023-00655-2 (PMC10486035; doi:10.1186/s13034-023-00655-2)
Supplement: Supplementary file 1 — Supplementary Material 1 [file 13034_2023_655_MOESM1_ESM.docx]

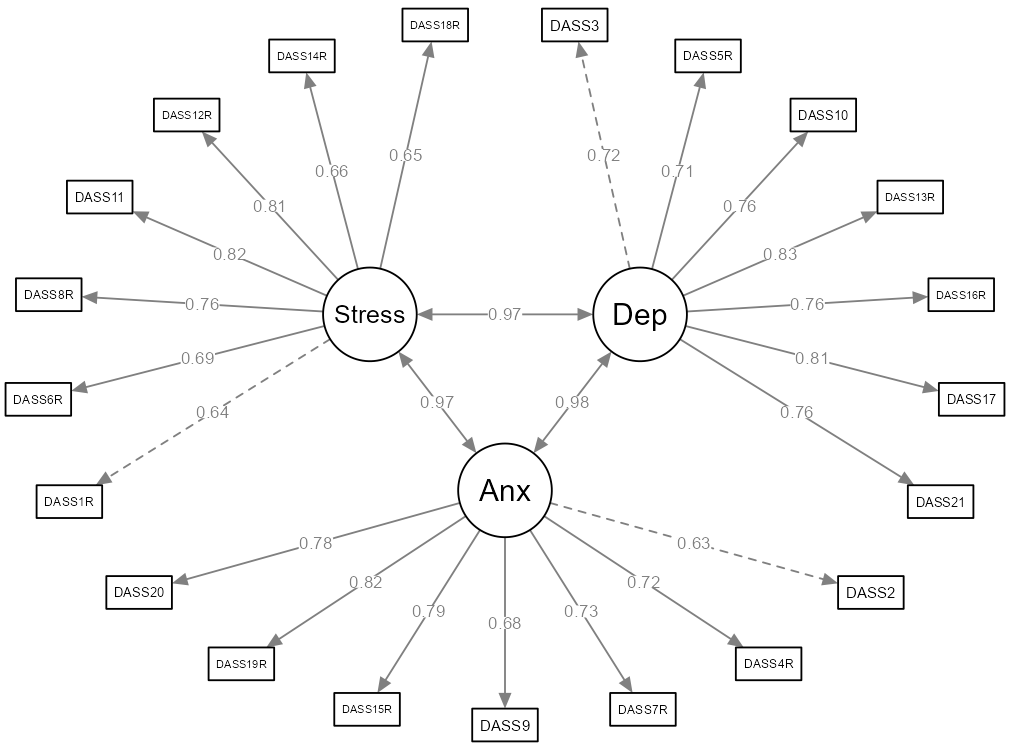

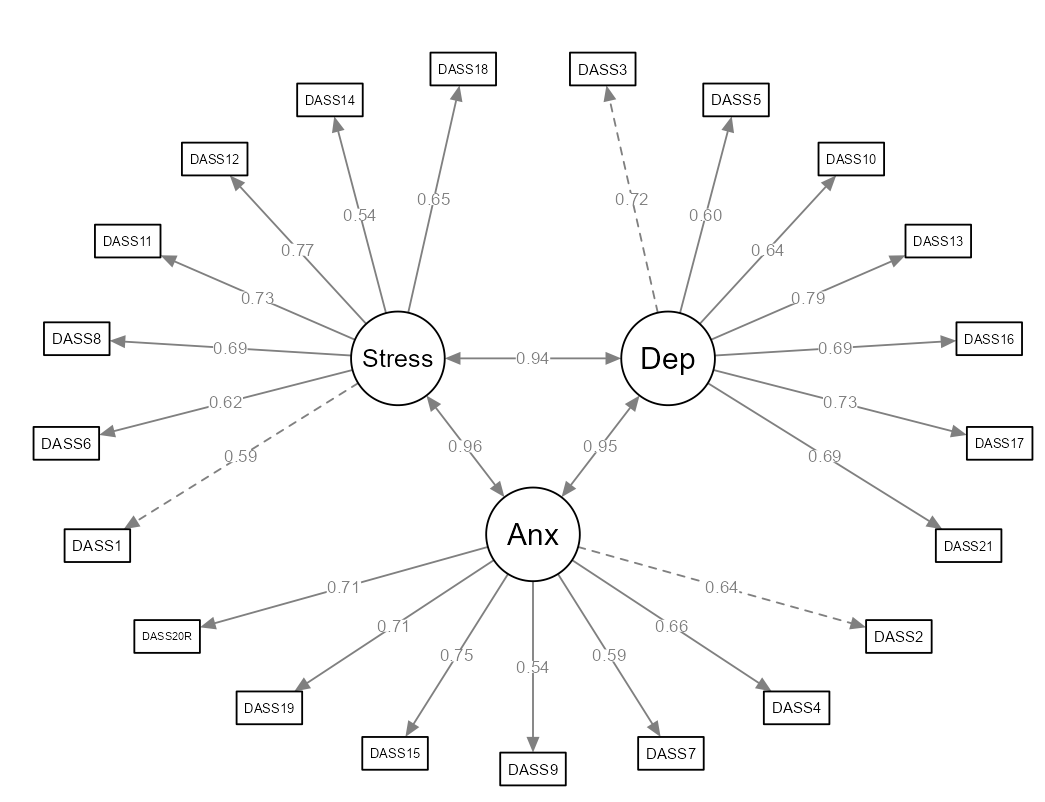


| Primary school students |  | Middle school students |
| --- | --- | --- |

Dep = Depression; Anx = Anxiety.

Figure S1 DASS-21 among primary and middle school students


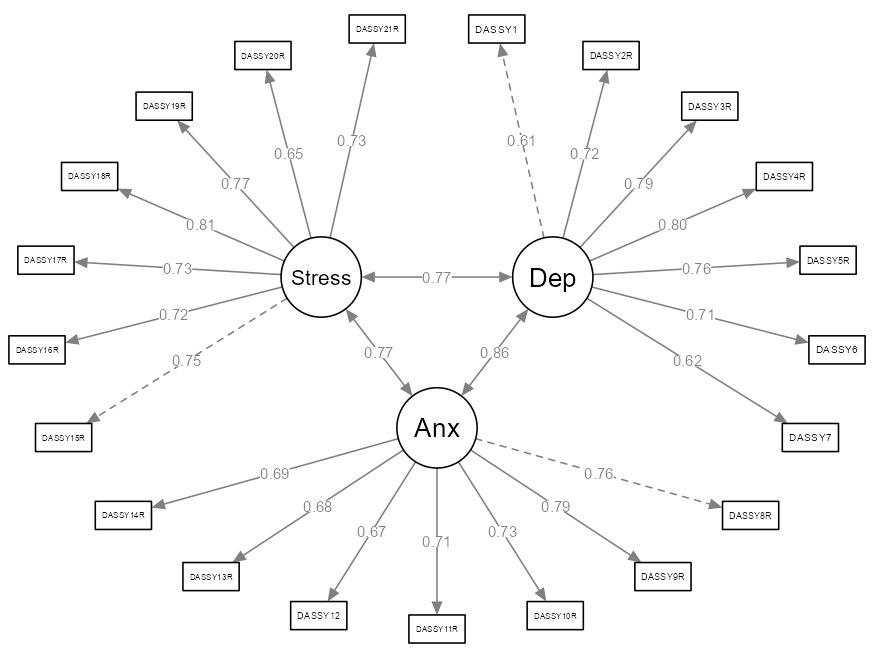

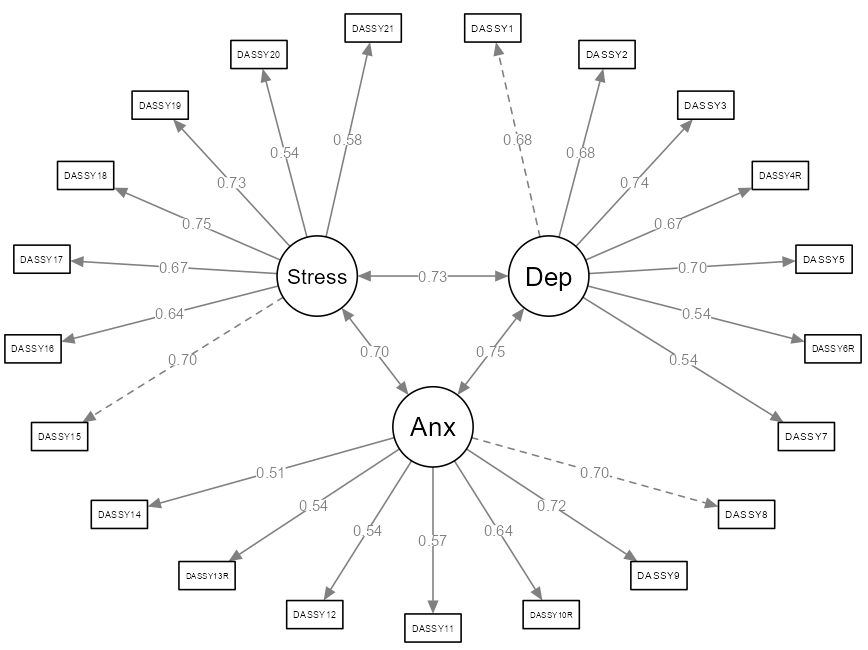


| Primary school students |  | Middle school students |
| --- | --- | --- |

Dep = Depression; Anx = Anxiety.

Figure S2 DASS-Y among primary and middle school students


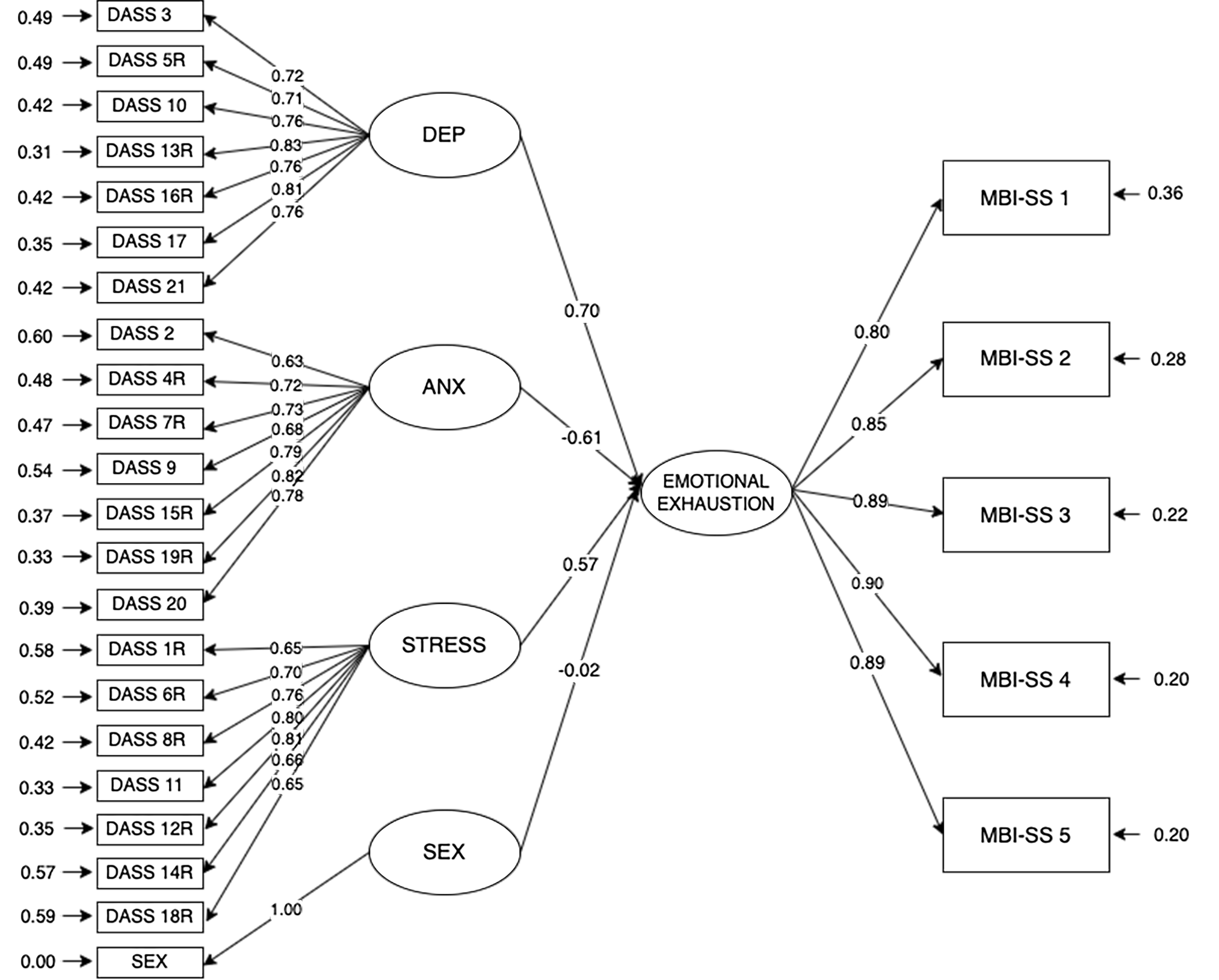

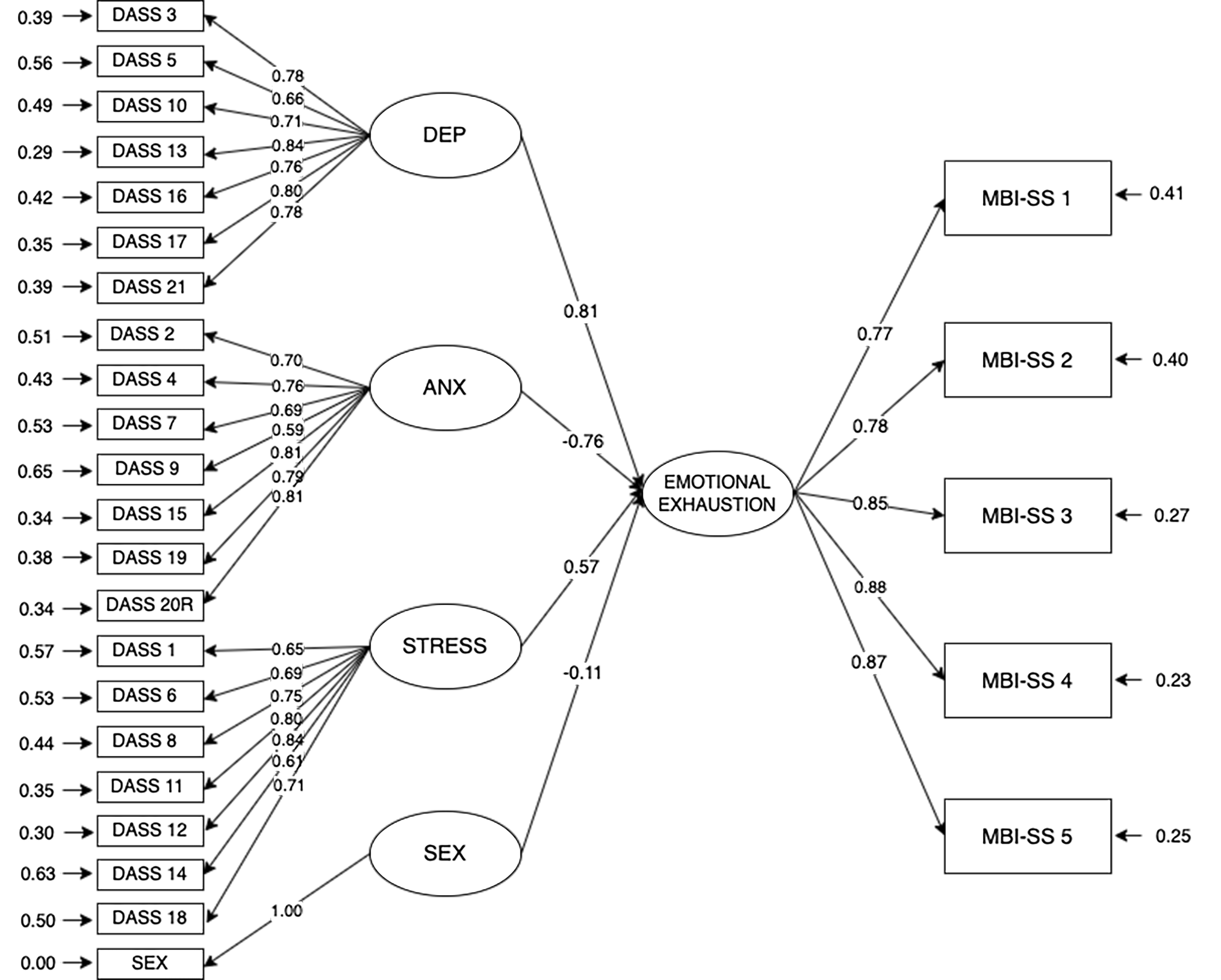


| Primary school students |  | Middle school students |
| --- | --- | --- |

DEP = Depression; ANX = Anxiety.

Figure S3 DASS-21 among primary and middle school students


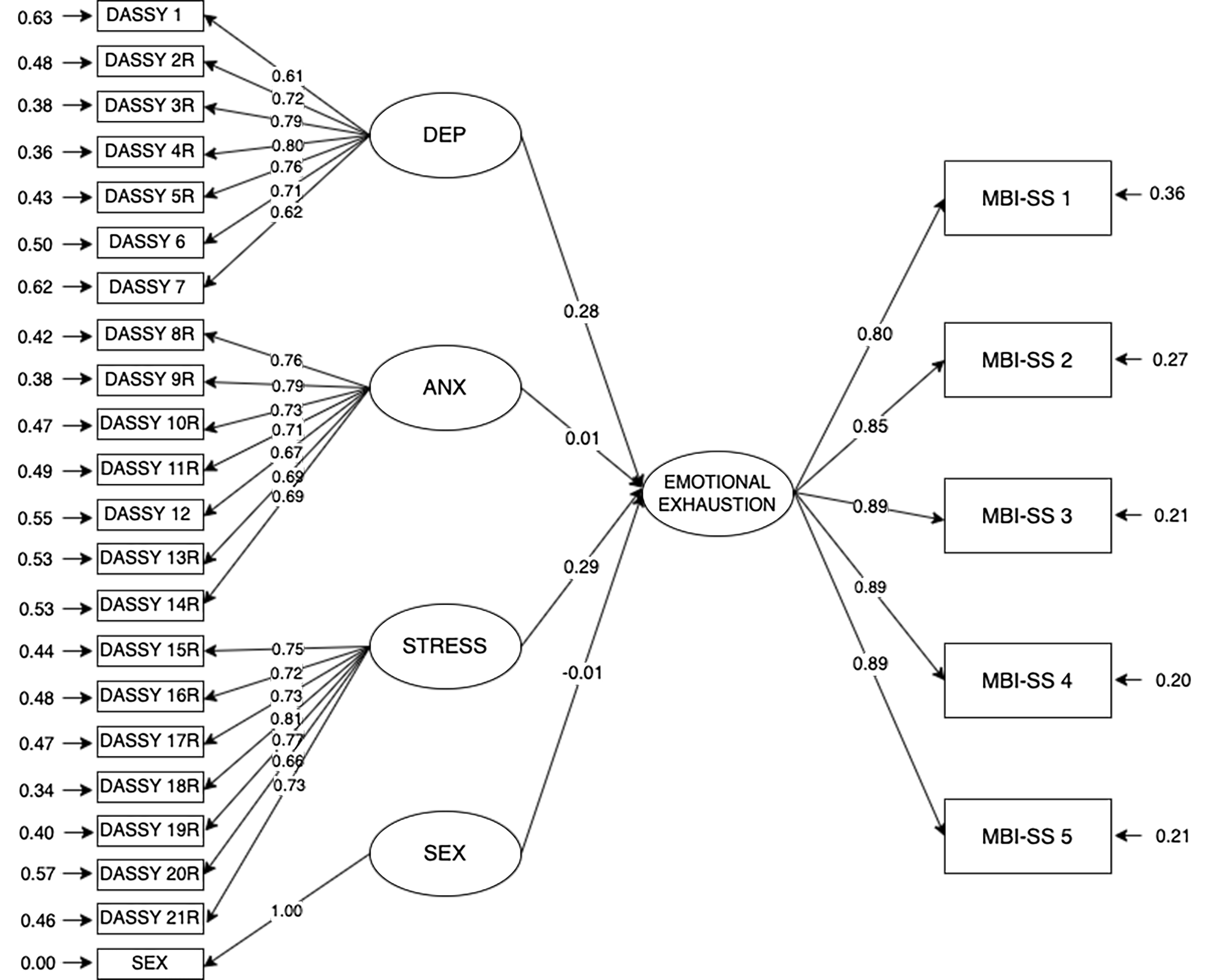

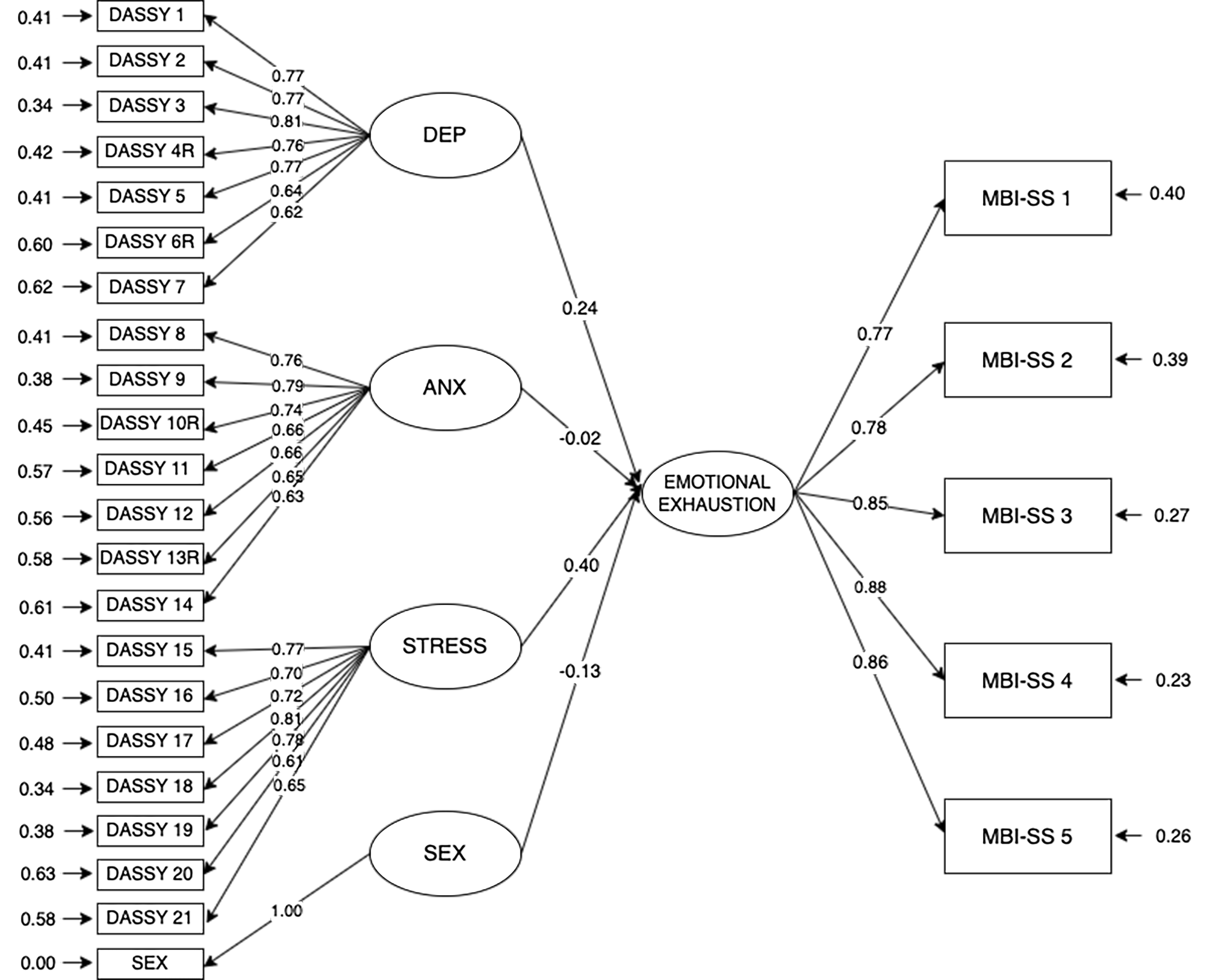


| Primary school students |  | Middle school students |
| --- | --- | --- |

DEP = Depression; ANX = Anxiety.

Figure S4 DASS-Y among primary and middle school students

Table S1. The original English version & Chinese version of DASS-Y

| The original English version | The Chinese version |
| --- | --- |
| 1. I hated my life. | 1. 我讨厌我的生活。 |
| 2. I hated myself. | 2. 我讨厌我自己。 |
| 3. I felt that life was terrible. | 3. 我觉得生活很糟糕。 |
| 4. I felt like I was no good. | 4. 我觉得我没用。 |
| 5. I could not stop feeling sad. | 5. 我一直感觉伤心。 |
| 6. I did not enjoy anything. | 6. 我什么都没有享受（到）。 |
| 7. There was nothing nice I could look forward to. | 7. 我没有什么期待的（好事）。 |
| 8. I felt like I was about to panic. | 8. 我感到快恐慌了。 |
| 9. I felt terrified. | 9. 我感到恐惧。 |
| 10. I felt scared for no good reason. | 10. 没有什么原因，（但）我感到害怕。 |
| 11. I could feel my heart beating. | 11. 即使没有做任何剧烈运动，我也能感觉到我的心跳非常快。 |
| 12. I had trouble breathing. | 12. 我不做运动、也没有生病的时候，我也呼吸困难。 |
| 13. My hands felt shaky. | 13. 我感觉手发抖。 |
| 14. I felt dizzy, like I was about to faint. | 14. 我感觉头晕，好像要晕倒了。 |
| 15. I got upset about little things. | 15. 因为一些小事，我感到烦恼。 |
| 16. I was easily irritated. | 16. 我很容易被激怒。 |
| 17. I found myself over-reacting to situations. | 17. 我发现自己对事情会反应过度。 |
| 18. I was easily annoyed. | 18. 我很容易感觉烦躁。 |
| 19. I was stressing about lots of things. | 19. 我对很多事情都感到有压力。 |
| 20. I got annoyed when people interrupted. . . | 20. 当有人打断我，我觉得难以忍受。 |
| 21. I found it difficult to relax. | 21. 我发现很难放松。 |

Table S2. The proportion of clinical-level emotional symptoms for primary school students and middle School Students

| **Primary School Students** | | | | | | |
| --- | --- | --- | --- | --- | --- | --- |
|  | Depression | | Anxiety | | Stress | |
|  | DASS-21 | DASS-Y | DASS-21 | DASS-Y | DASS-21 | DASS-Y |
| Normal | 1289 (85.5%) | 1362 (90.4%) | 1234 (81.9%) | 1401 (93.0%) | 1411 (93.6%) | 1477 (98.0%) |
| Mild | 63 (4.2%) | 94 (6.2%) | 43 (2.9%) | 69 (4.6%) | 54 (3.6%) | 13 (0.9%) |
| Moderate | 121 (8.0%) | 37 (2.5%) | 149 (9.9%) | 21 (1.4%) | 27 (1.8%) | 12 (0.8%) |
| Severe | 22 (1.5%) | 11 (0.7%) | 41 (2.7%) | 10 (0.7%) | 12 (0.8%) | 3 (0.2%) |
| Extremely severe | 12 (0.8%) | 3 (0.2%) | 40 (2.7%) | 6 (0.4%) | 3 (0.2%) | 2 (0.1%) |
| McNemar-Bowker Test: *χ*^2^ (*p*-value) | 81.16 (<0.01) |  | 200.86 (<0.01) |  | 52.60 (<0.01) |  |
| **Middle school Students** | | | | | | |
| Normal | 626 (55.3%) | 890 (78.7%) | 519 (45.9%) | 928 (82.1%) | 826 (73.0%) | 1026 (90.7) |
| Mild | 174 (15.4%) | 138 (12.2%) | 126 (11.1%) | 120 (10.6%) | 137 (12.1%) | 45 (4.0) |
| Moderate | 212 (18.7%) | 79 (7.0) | 242 (21.4%) | 66 (5.8%) | 102 (9.0%) | 35 (3.1) |
| Severe | 68 (6.0%) | 16 (1.4) | 99 (8.8%) | 10 (0.9%) | 47 (4.2%) | 15 (1.3) |
| Extremely severe | 51 (4.5%) | 8 (0.7) | 145 (12.8%) | 7 (0.6%) | 19 (1.7%) | 10 (0.9) |
| McNemar-Bowker Test: *χ*^2^ (*p*-value) | 418.36 (<0.01) |  | 531.07 (<0.01) |  | 177.78 (<0.01) |  |
